# Engineering and application of multiepitope recombinant proteins to enhance resistance to *Botrytis cinerea* in tomatoes: a new paradigm for creating plant immune activators

**DOI:** 10.3389/fpls.2025.1499777

**Published:** 2025-02-17

**Authors:** Xiaxia Man, Huang You, Zhiqiang Cheng, Junhao Li, Dunchao Yao, Haofeng Wang, Zhihong Diao, Xiaosong Yu, Wei Wu, Cheng Zhou, Yan Huang, Jinbo Shen, Xiaohong Zhuang, Yi Cai

**Affiliations:** ^1^ College of Life Sciences, Sichuan Agricultural University, Yaan, Sichuan, China; ^2^ Chengdu Lusyno Biotechnology Co., Ltd, Chengdu, Sichuan, China; ^3^ State Key Laboratory of Subtropical Silviculture, Zhejiang A&F University, Hangzhou, Zhejiang, China; ^4^ Centre for Cell and Developmental Biology, State Key Laboratory of Agrobiotechnology, School of Life Sciences, The Chinese University of Hong Kong, Hong Kong, Hong Kong SAR, China

**Keywords:** recombinant protein, plant elicitor, tomato immunity, *Botrytis cinerea* resistance, TMV

## Abstract

Plant elicitors have emerged as key agents in effectively invoking immune responses across various plant species, gaining attention for their role in sustainable agricultural protection strategies. However, the economic utility of peptide elicitors such as flg22, flgII-28, and systemin is limited when considering broader agricultural applications. This study introduces a novel recombinant protein, SlRP5, which integrates five active epitopes—flg22, csp22, flgII-28, SIPIP1, and systemin—to activate immune responses and significantly enhance resistance to *Botrytis cinerea* in tomatoes (*Solanum lycopersicum*). SIRP5 significantly induced reactive oxygen species (ROS), MAPK activation, and callose deposition in tomato leaves during *in vitro* experiments. Transcriptomic analysis revealed that SlRP5 more effectively activated key immune-related pathways compared to traditional peptides, upregulating critical genes involved in calcium-binding proteins and phenylpropanoid biosynthesis. In further *in vivo* experiments, SlRP5 alleviated *B. cinerea*-induced membrane damage by reducing MDA and REC levels, while simultaneously enhancing the activities of antioxidant enzymes such as SOD, CAT, and POD, thereby mitigating the excess ROS generated by infection. Additionally, SlRP5 elicited significant immunological effects in tobacco and eggplant, characterized by ROS bursts and callose deposition. It amplified tobacco’s resistance to TMV and mitigated *B. cinerea*-induced damage in eggplant. These findings underscore the substantial potential of SlRP5 as a plant immune activator, integrating multiple immune-eliciting peptides, and offering a novel strategy for cultivating new biopesticides that can induce immune responses and heighten disease resistance in various crops.

## 1 Introduction

Plants have developed a sophisticated and effective immune system, which plays a crucial role in defending against potential pathogens attacks. This system operates by initiating an array of defense mechanisms, both locally and systemically, to prevent or manage infections. Pattern recognition receptors (PRRs) on plant cell surfaces detect conserved molecular patterns associated to microbial infections, initiating a cascade of early defense responses. These include the production of reactive oxygen species (ROS), calcium ion (Ca^2+^) influx, and the activation of Mitogen-Activated Protein Kinase (MAPK) signaling pathways. These initial defenses trigger subsequent immune responses, such as callose deposition on the cell wall, upregulation of immune-related genes, and increased activity of enzymes associated with disease resistance, thereby strengthening the plant’s overall defense mechanisms. Through these intricate, sequential responses, plants effectively equip themselves to combat pathogen invasions.

Plant elicitors, which can activate plant immune responses without directly affecting pathogens, have garnered significant attention in recent years (Greco et al., 2024). Among these, peptide-based elicitors are the most extensively studied type (Del Corpo et al., 2024). Various immune activators have been identified from bacteria, fungi, oomycetes, viruses, and plants. Flg22 is a well-studied peptide-based elicitor known to trigger a series of rapid responses, including Ca^2+^ influx, ROS production, and ethylene synthesis (Felix et al., 1999; Gómez-Gómez et al., 1999; Zipfel et al., 2004; Boller and Felix, 2009; Jelenska et al., 2017). In certain Solanaceae plants, the secondary sensing system FLS3 recognizes flgII-28, triggering immune responses and enhancing disease resistance. Conserved structural components, such as xup25, Pep-13, csp22, nlp20, and SsCut from pathogens, have also been demonstrated to induce host immune responses. Pep-13, a peptide derived from the soybean pathogen *Phytophthora sojae*, effectively triggers immune responses in parsley and potato plants. Csp22, a polypeptide elicitor from the bacterium *Ralstonia solanacearum*, is specifically recognized by Solanaceae plants and has been shown to inhibit the growth of bacterial wilt in tomatoes. Nlp20, which is widely found in bacteria, fungi, and oomycetes, plays a critical role in plant immune responses during pathogen infections. Furthermore, plants can also trigger immune responses through signaling molecules released in response to physical damage or pest attacks. For example, the application of synthetic peptides Atpep1 and PIP1 has been shown to enhance *Arabidopsis* resistance against *Pseudomonas syringae* and *Fusarium oxysporum*. Systemin, an 18-amino acid polypeptide isolated from wounded tomato leaves, activates systemic immune responses in Solanaceae plants, thereby enhancing resistance to fungal pathogens such as *Botrytis cinerea* and *Alternaria* spp., as well as to aphids (*Macrosiphum euphorbiae*).

As potential candidates for biological control agents, plant immune elicitors enhance plant resistance to diseases without causing adverse effects on the environment or nontarget beneficial organisms. The development of plant immune inducers has become a key trend in the global biopesticide industry, rapidly emerging as a strategic sector with significant potential. For instance, harpin, a protein extracted from Gram-negative bacteria, has been shown to increase crop yields and enhance resistance to various diseases and aphid infestations. The harpin-based biopesticide messenger has been approved for use on crops such as tomatoes, tobacco, and rapeseed. Additionally, the plant immune-inducing protein PeaT1 has shown efficacy in enhancing viral resistance and promoting plant growth. Based on research into PeaT1, the Chinese Academy of Agricultural Sciences has developed a commercial plant immune inducer named ATaiLin, which features PeaT1 as its main component. Furthermore, natural substances like chitosan have also exhibited significant plant immune elicitation capabilities and are now widely applied in the biopesticide industry.

Tomato (*Solanum lycopersicum*) is a widely cultivated economic crop globally, but it is highly susceptible to infections by necrotrophic fungal pathogens, such as *B. cinerea*, which lead to significant reductions in both yield and quality. Despite the common use of chemical fungicides for disease management, their extensive application has raised significant concerns regarding environmental pollution and the emergence of pathogen resistance. Consequently, the pursuit of new, environmentally friendly biopesticides has become a crucial priority in the quest for sustainable agricultural practices. Plant elicitors, as exemplars of innovative biopesticides that harness plant defense mechanisms, exhibit significant potential for application. However, the development of recombinant immunity-inducing proteins specifically targeting tomatoes has been scarcely documented.

In this study, we designed a recombinant protein, SlRP5, by combining the active epitopes of flg22, csp22, flgII-28, SlPIP1, and systemin, based on their activity to induce PAMP-induced immunity (PTI), with the goal of enhancing immune responses in tomatoes. Our evaluation of SlRP5’s immune-inducing and disease-resistance capabilities revealed that it effectively activated immune responses across various Solanaceae plants, providing them with enhanced resistance against pathogens. This discovery provides a theoretical foundation for the future development of biopesticides and offers new possibilities for achieving safer and more efficient plant disease management strategies.

## 2 Materials and methods

### 2.1 Plant materials

Tomato (*Solanum lycopersicum* cv. Jinpeng 1) seeds were purchased from Shanxi Jinpeng Seedling Co. Ltd. (Xian, China). Eggplant (*Solanum melongena* cv. Yuqie 5) seeds were obtained from Chongqing Keguang Seedling Co. Ltd. (Chongqing, China). *Nicotiana tabacum* cv. Sansun and *N. tabacum* cv. NC89 seeds were preserved in our laboratory, with NC89 used exclusively for the propagation of Tobacco Mosaic Virus (TMV). All plants used in this study were grown in an environmentally controlled growth room at 22°C–25°C, 60%–80% relative humidity, with a 16-h photoperiod.

### 2.2 Synthesis of elicitors and purification of recombinant protein

Peptides were synthesized at > 95% purity by GenScript (Nanjing, China). The sequence of all peptides are described in **Supplementary Table 1**. Peptides were prepared as 5 mM stock solutions in dH_2_O and diluted in dH_2_O prior to use. The SlRP5 coding sequence was synthesized by Sangon Biotech (Shanghai, China) and cloned into the *pET-28a* vector, which was then transformed into *Escherichia coli* BL21(DE3). For protein expression, a single colony was inoculated into 5 mL of LB medium containing 50 μg mL^−1^ kanamycin and cultured overnight at 37°C with shaking at 200 rpm. The overnight culture was transferred to 1,000 mL of fresh LB medium (1:100 dilution) and grown until the OD600 reached 0.6–0.8. Protein expression was induced by adding 1 mM IPTG, and the culture was incubated at 37°C for 6 h with shaking. The cells were harvested by centrifugation at 6,000×*g* for 10 min at 4°C and resuspended in PBS buffer. The resuspended cells were lysed using an ultrasonic cell disruptor, and the target protein was subsequently collected and purified. The protein was purified using HisSep Ni-NTA Agarose Resin 6FF and quantified with a BCA Protein Quantification Kit, both from Yeasen Biotechnology (Shanghai, China). For experimental convenience, the purified SlRP5 protein was concentrated and desalted using ultrafiltration centrifuge tubes (Membrane Solutions, Shanghai, China) and then dissolved in dH_2_O.

### 2.3 Immune response detection

The production of ROS in leaves was quantified using a previously described method (Kunze et al., 2004). Briefly, samples were placed in a 96-well plate prefilled with a reaction mixture containing 2 μg mL^−1^ of horseradish peroxidase and 200 μM of luminol-20. After the addition of 1 μM peptide or recombinant protein, luminescence emitted from the reaction was measured using a microplate reader. Callose deposits were stained as previously described (Clay et al., 2009). Briefly, plant leaves were treated with 1 μM peptides or recombinant protein for 16–18 h, followed by fixation in FAA solution (75% ethanol, 25% acetic acid) for 6 h. The leaves were then cleared in 50% ethanol until they became transparent. Finally, samples were incubated in the staining solution (0.01% aniline blue in 67 mM K_2_HPO_4_, pH 9.5) in the dark for 1 h and observed under a UV fluorescence microscope. The relative intensity of callose deposition was quantified according to a previously described method (Luna et al., 2011). The accumulation of H_2_O_2_ was visualized using 3,3′-diaminobenzidine (DAB), while O{sp}2•−{/sp} was detected using nitroblue tetrazolium (NBT) (Thordal-Christensen et al., 1997; Wohlgemuth et al., 2002). Briefly, detached leaves were incubated in 1 μM peptide or recombinant protein solution for 4 h, followed by staining with DAB or NBT for 12 h. The leaves were then decolorized at 95°C using a solution of 60% ethanol, 20% glycerol, and 20% acetic acid. The stained areas were quantified using ImageJ. Western blots to determine MAPK activation were performed as previously described (Flury et al., 2013). Detached plant leaves were treated with 1 μM peptide or recombinant protein for 15 min. After extracting total protein, active MPK6 and MPK3 were detected using a phospho-p44/p42 MAPK antibody from Cell Signaling Technology (Danvers, MA, USA) via immunoblot analysis. Unless otherwise stated, all peptides or recombinant proteins used in the immunoassays in this experiment were at a concentration of 1 μM.

### 2.4 Pathogen inoculation

The *B. cinerea* was cultured on potato dextrose agar (PDA) medium at 22°C for 3 to 4 weeks. The hyphae were soaked with 5 mL of dH_2_O and then scraped to release sporangia. Plates containing the sporangia solution were oscillated for 10 min to release zoospores, which were then filtered through four layers of gauze to remove the hyphae. Plant leaves were sprayed with a pathogen spore suspension at a concentration of 2 × 10^6^ spores L^−1^ (Zhang et al., 2018). To quantify the lesion area, the inoculated area was photographed, and the lesion size was measured using ImageJ. Disease severity was classified according to the standard GB/T 17980.28-2000 (China). The preparation and inoculation of TMV were performed as previously described (Wang et al., 2016). NC89 leaves infected with TMV were ground with a small amount of quartz sand and 1× PBS buffer. The homogenate was then filtered through four layers of gauze to obtain the crude virus extract. The concentration of the crude virus extract was adjusted to 1:40 (g mL^−1^) for Sansun inoculation. After inoculation, the plants were cultivated until the control group was fully symptomatic. The number of necrotic spots on the leaves was then photographed and recorded. Unless specified otherwise, a concentration of 3 μM was used in the experiments to induce plant pathogen resistance by SlRP5.

### 2.5 Enzyme activity assays

To determine the activities of superoxide dismutase (SOD), peroxidase (POD), and catalase (CAT), enzyme extracts were prepared. SOD and POD extracts were obtained by homogenizing 0.2 g of fresh leaves in 2 mL of 0.1 M phosphate buffer (pH 7.8), while CAT extracts were prepared by homogenizing 0.5 g of fresh leaves in 2 mL of 0.1 M phosphate buffer (pH 6.0). The homogenates were filtered through muslin cloth and centrifuged at 10,000×*g* for 20 min at 4°C. The supernatants were collected as enzyme extracts. SOD activity was measured based on its ability to inhibit the photochemical reduction of NBT (Agarwal and Pandey, 2004). The reaction mixture consisted of 10 µL of enzyme extract and 90 µL of SOD reaction buffer, which contained 0.1 M phosphate buffer, 130 mM methionine, 750 µM NBT, 100 µM EDTA-Na_2_, and 20 µM riboflavin. The mixture was added to a 96-well plate and incubated under fluorescent light for 10 min, after which absorbance was measured at 560 nm. POD activity was determined by monitoring the increase in absorbance at 470 nm due to the oxidation of guaiacol (Erdem et al., 2015). The reaction mixture consisted of 10 µL of enzyme extract and 90 µL of POD reaction buffer, which contained 0.1 M phosphate buffer, 20 mM guaiacol, and 10 mM H_2_O_2_. The linear increase in absorbance at 470 nm was used to calculate POD activity. CAT activity was measured by monitoring the decrease in absorbance at 240 nm due to the decomposition of H_2_O_2_ (Havir and McHale, 1987). The reaction mixture consisted of 0.1 mL of enzyme extract and 2.5 mL of CAT reaction buffer containing 30 mM H_2_O_2_. The enzyme activity was calculated using the extinction coefficient of H_2_O_2_.

### 2.6 Lipid peroxidation and electrolyte leakage

Malondialdehyde (MDA) content was measured using the thiobarbituric acid (TBA) method. Fresh leaves (0.2 g) were homogenized in 2 mL of 5% (w/v) trichloroacetic acid (TCA) and centrifuged at 9,000×*g* for 20 min. An equal volume of 10% TCA containing 0.67% TBA was added to the supernatant, and the mixture was boiled for 30 min before being cooled in an ice bath for 5 min. The absorbance of the supernatant was measured at 450 nm, 532 nm, and 600 nm. Electrolyte leakage was assessed by measuring relative electrolytic conductivity (REC) (Feng et al., 2013). Plant material (0.5 g) was placed in 30 mL of deionized water at room temperature for 12 h to measure initial conductivity (R1). The samples were then boiled for 30 min, cooled to room temperature, and the final conductivity (R2) was measured. Deionized water conductivity (R0) was used as a blank. REC was calculated using the following formula (Equation 1):


(1) (R1−R0)/(R2−R0)×100%

### 2.7 Determination of chlorophyll

Total chlorophyll was extracted from 0.2 g of leaves by placing them in a centrifuge tube containing 10 mL of 95% ethanol. The samples were kept in the dark until the green color completely faded. Subsequently, 100 μL of the extract was added to a 96-well plate. The absorbance at 665 nm and 649 nm, corresponding to chlorophyll *a* and chlorophyll *b*, was measured according to previously described methods (Wellburn and Lichtenthaler, 1984). Chlorophyll fluorescence of PSII was analyzed at room temperature using the Imaging-PAM M-Series Chlorophyll Fluorometer (Heinz-Walz Instruments, Effeltrich, Germany) following previously described methods. Before measuring chlorophyll fluorescence, tomato samples were dark-adapted for 30 min. Imaging and calculation of the effective quantum yield [Y(II)] were performed according to the method described by Maxwell and Johnson (2000).

### 2.8 RNA isolation and qPCR analysis

Total RNA extracted from tomato leaves using the MolPure^®^ Plant RNA Kit (Yeasen Biotechnology, Shanghai, China). Real-time fluorescence quantification was performed with the ChamQ Universal SYBR qPCRMaster Mix (Vazyme, Nanjing, China). Samples were normalized using the comparative CT method, with transcription levels of target genes aligned to the expression of tomato *SlActin* (Solyc03g078400). Primers used in real-time PCR are listed in **Supplementary Table 2**.

### 2.9 Transcriptome analysis

Detached leaves were treated with 3 μM recombinant SlRP5 and flgII-28 for 4 h, then quickly frozen in liquid nitrogen and collected for transcriptome analysis. Each treatment group included three biological replicates. Sequencing of qualified RNA samples was performed by Majorbio Biomedical Technology Co. Ltd. (Shanghai, China). Clean reads from each sample were aligned to the reference tomato genome (Tomato Genome SL4.0, https://data.jgi.doe.gov/refine-download/phytozome.com.cn) using HISAT2. Gene expression data were represented as fragments per kilobase of transcript per million mapped reads (FPKM). Differentially expressed genes (DEGs) were analyzed using DESeq2 with the following parameters: |log_2_(FC) | > 1, significance *p*-value < 0.05. GO and KEGG pathway analyses were conducted using Majorbio online tools (http://www.majorbiogroup.com/). Volcano plots of DEGs were created using the ggplot2 package, and heatmaps were generated by the online platform (https://www.bioinformatics.com.cn).

## 3 Results

### 3.1 Flg22, CSP22, systemin, SlPIP1, and FlgII-28 effectively trigger immune responses in tomato

**Figure 1:**
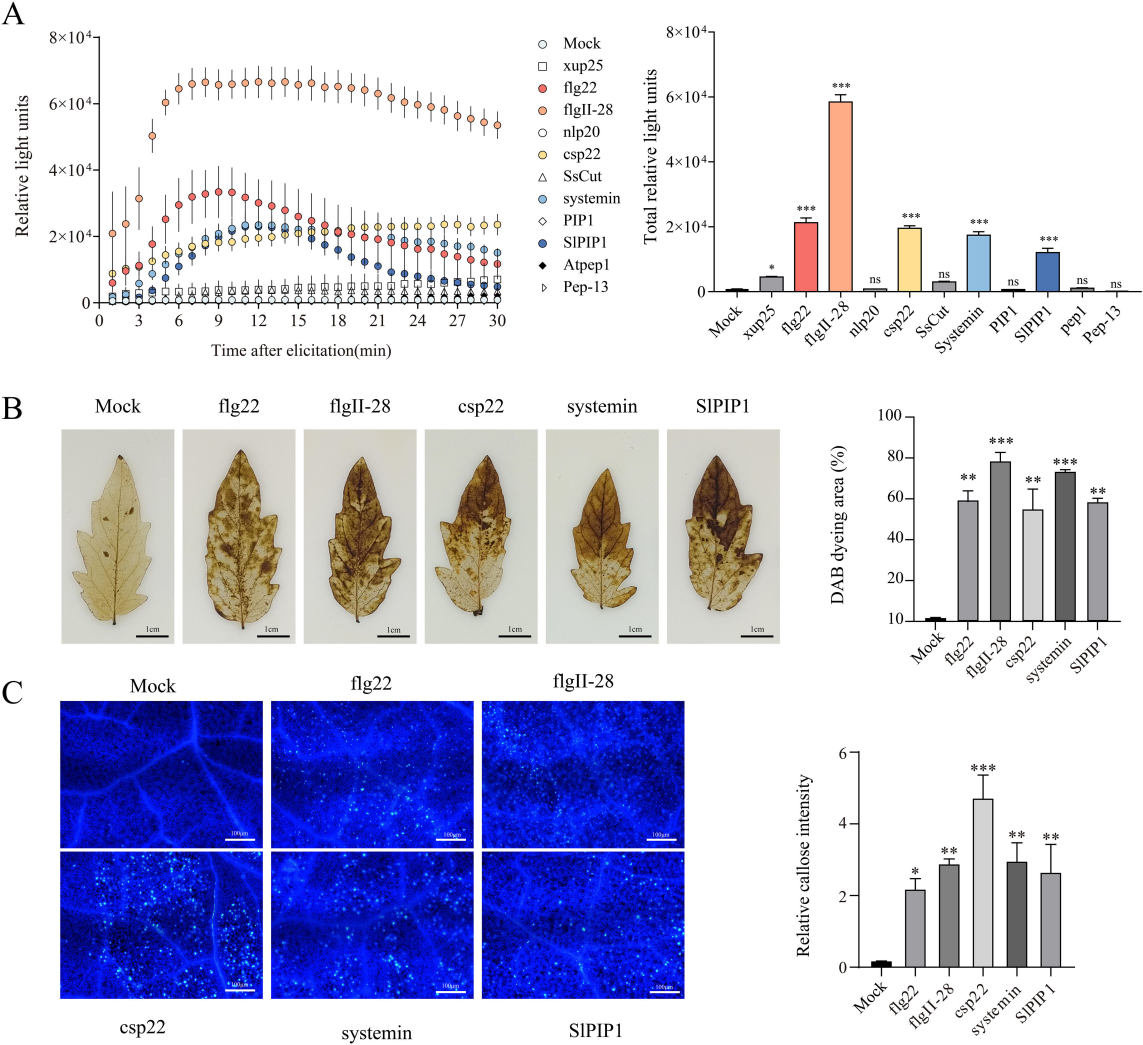
Evaluation of peptides that activate immune responses in tomatos. **(A)** Kinetics of ROS burst induced by different elicitors. The left panel shows the dynamics of ROS production within 30 minutes after elicitation, and the right panel shows the total ROS production. The values are means ± SEM (one-way ANOVA, *n* = 4). **(B)** Detection of hydrogen peroxide accumulation using DAB staining. The right panel shows the analysis of DAB staining using ImageJ software. The values are means ± SEM (one-way ANOVA, *n* = 3). **(C)** Detection of callose deposition using aniline blue staining. The right panel shows the quantification of relative callose intensity. The values are means ± SEM (one-way ANOVA, *n* = 20). Asterisks indicate a significant difference from the Mock (dH_2_O) at *p < 0.05, **p < 0.01, and ***p < 0.001. ns indicates no significant difference.

To identify peptides potentially involved in tomato immune responses, we tested 12 peptides, including flg22, flgII-28, Atpep1, Pep-13, nlp20, csp22, SsCut, xup25, systemin, PIP1, and SlPIP1. The activity of these immune elicitors was assessed using the rapid PAMP-induced ROS burst (Mukhi et al., 2017; Wei et al., 2020). Within 30 min, the production of ROS induced by flg22, flgII-28, csp22, Systemin, and SlPIP1 was found to be higher in 4-week-old tomato plants (**Figure 1A**). DAB staining indicated that treatments with flg22, flgII-28, csp22, systemin, and SlPIP1 significantly increased H_2_O_2_ accumulation in tomato leaves, as evidenced by the appearance of reddish-brown discolorations. Among these, flgII-28 induced the highest H_2_O_2_ levels, with staining coverage exceeding 70%, followed by csp22, flg22, and systemin, which exhibited moderate levels. SlPIP1 displayed relatively lower induction, with staining intensity around 50% (**Figure 1B**). Callose deposition is also an important indicator of plant immune activity (Luna et al., 2011). We observed that treatment with five peptides significantly increased callose deposition in tomato leaves, whereas no such increase was detected in the mock-treated controls (**Figure 1C**). These findings suggest that flg22, flgII-28, csp22, systemin, and SlPIP1 effectively activate immune responses in tomatoes.

### 3.2 Expression and purification of the SlRP5

**Figure 2:**
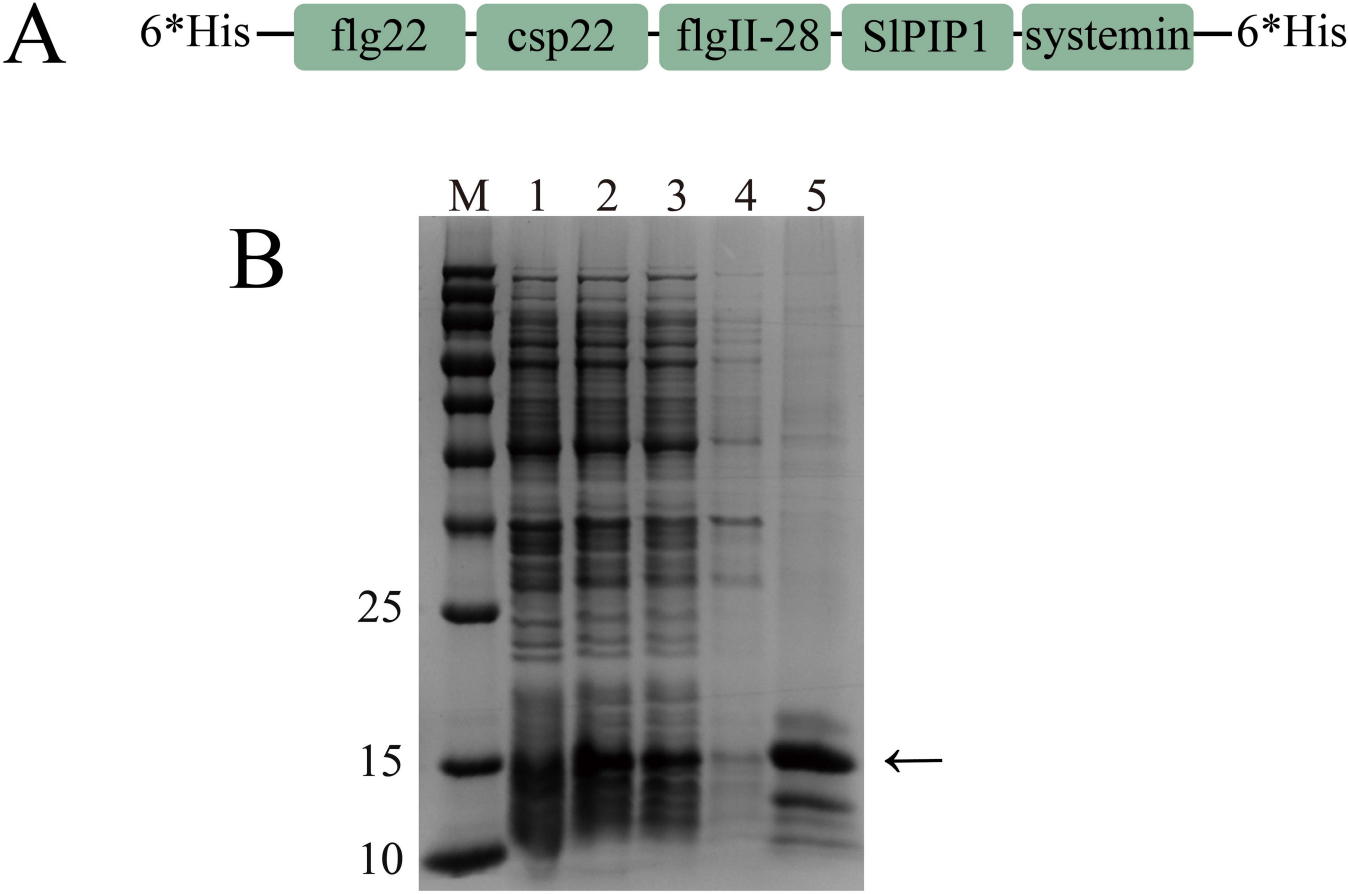
Expression and purification of recombinant protein SlRP5. **(A)** Schematic representation of the structure of recombinant protein SlRP5. The schematic illustrates five peptides linked by GGG sequences, with 6*His tags at the N-terminal and C-terminal. **(B)** Prokaryotic expression and purification of recombinant protein SlRP5. M, protein molecular weight marker; 1, before IPTG induction; 2, after IPTG induction; 3, supernatant protein; 4, pellet protein; 5, purified protein.

The prohibitive production costs of using peptides as primary components in biopesticides limit their practicality for agricultural applications. To mitigate this issue, we engineered a recombinant protein by concatenating flg22, csp22, systemin, SlPIP1, and flgII-28 (**Figure 2A**). We developed a *pET28a(+)-SlRP5* recombinant plasmid and expressed the recombinant protein in *Escherichia coli* BL21(DE3) as 6*His-SlRP5-6*His, following established protocols. After induction with 1 mM IPTG for 6 h, SlRP5 was successfully expressed in the supernatant. The protein was then purified using HisSep Ni-NTA Agarose Resin 6FF, yielding in a relatively pure form of SlRP5 (**Figure 2B**).

### 3.3 SlRP5 induces robust immune responses in tomato

**Figure 3:**
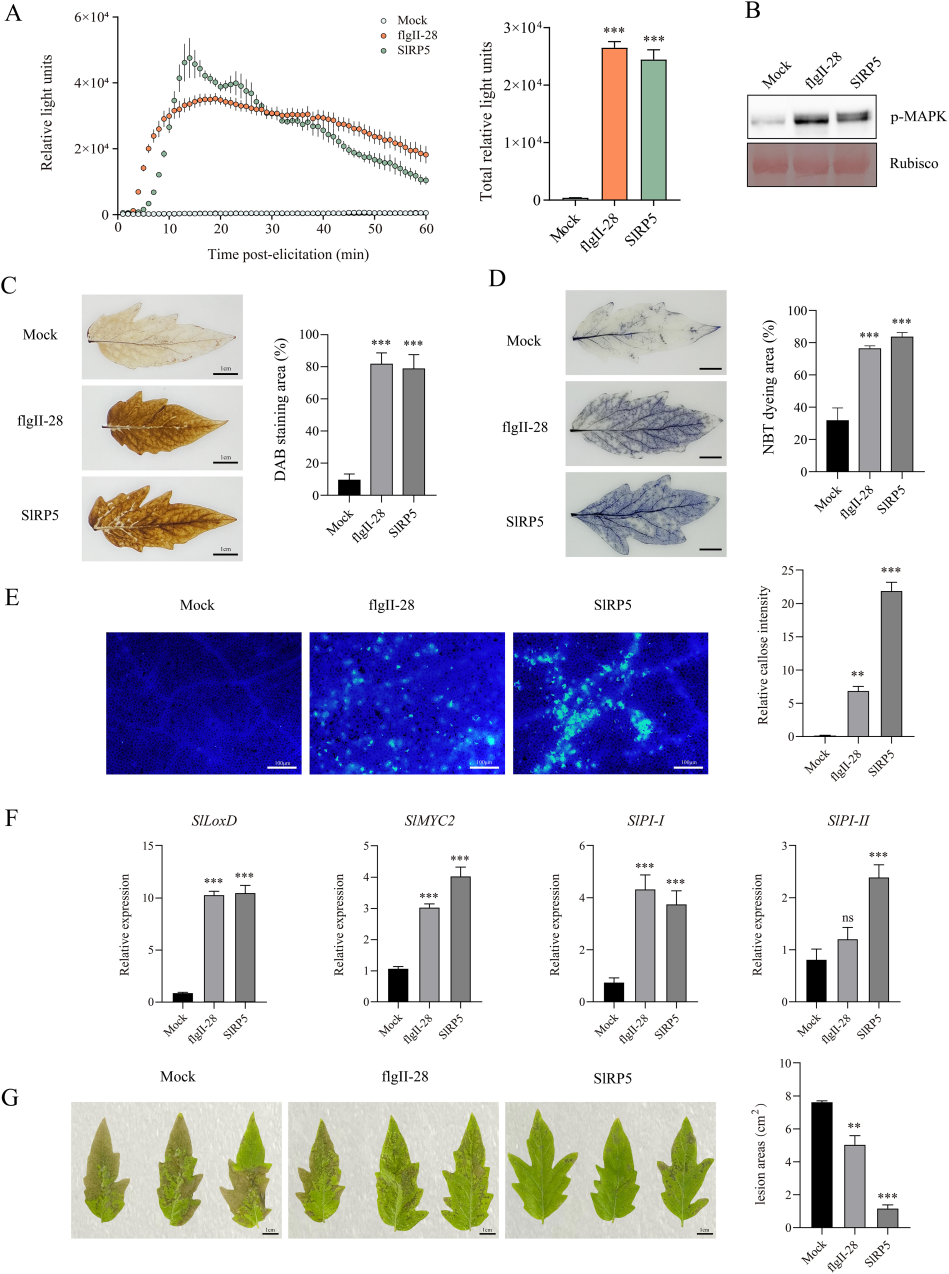
Immune Responses Induced by Recombinant Protein SIRPS in Tomato. **(A)** Recombinant protein SIRPS induces a burst of ROS in tomato. Data are presented as means ± SEM (one-way ANOVA, *n* = 4). **(B)** SIRP5 recombinant proteins trigger the activation of MAPK. **(C, D)** SIRP5 recombinant proteins induce the accumulation of H_2_O_2_ **(C)** and O^2^.− **(D)** in tomato. Data are presented as means ± SEM (one-way ANOVA, *n* = 3). **(E)** SIRP5 recombinant proteins induce callose deposition in tomato leaves. The right panel shows the quantification of relative callose intensity. Data are presented as means ± SEM (one-way ANOVA, *n* = 20). **(F)** Transcript levels of JA-mediated defense-related genes. Data are presented as means ± SEM (one-way ANOVA, *n* = 4). **(G)** Tomato leaves treated with SIRPS show fewer lesion areas. The right panel quantifies the lesion areas. Data are presented as means ± SEM (one-way ANOVA, *n* = 3). Asterisks indicate significant differences from the Mock (dH_2_O): **p < 0.01, ***p < 0.001. ns indicates no significant difference.

To evaluate the functional activity of the recombinant protein SlRP5, we analyzed its ability to induce immune responses in tomatoes. The results showed that treating leaf discs with 1 μM SlRP5 induced a significant ROS burst (**Figure 3A**), and MAPK activation was detected within 10 min via immunoblot analysis (**Figure 3B**; **Supplementary Figure 1**). DAB and NBT staining further confirmed that SlRP5 led to the accumulation of H_2_O_2_ and O{sp}2·−{/sp} in the leaves (**Figures 3C, D**). Additionally, callose deposition was assessed in tomato leaves, revealing that SlRP5 treatment induced significantly higher callose deposition compared to flgII-28 treatment (**Figure 3E**). In analyzing the expression patterns of JA signaling pathway-related genes, both SlRP5 and flgII-28 significantly induced the expression of LOXD, MYC2, and PI-I. However, flgII-28 treatment did not significantly increase PI-II expression (**Figure 3F**). Following *B. cinerea* inoculation, SlRP5 treatment triggered a more pronounced immune response than flgII-28 (**Figure 3G**). Although both SlRP5 and flgII-28 induced plant immune responses through the JA signaling pathway, SlRP5 was more effective, particularly in enhancing disease resistance post-*B. cinerea* inoculation. Collectively, these findings demonstrate that SlRP5 exhibits superior immune-inducing effects compared to flgII-28.

### 3.4 Transcriptomic changes in tomato triggered by SlRP5 and flgII-28 treatment

**Figure 4:**
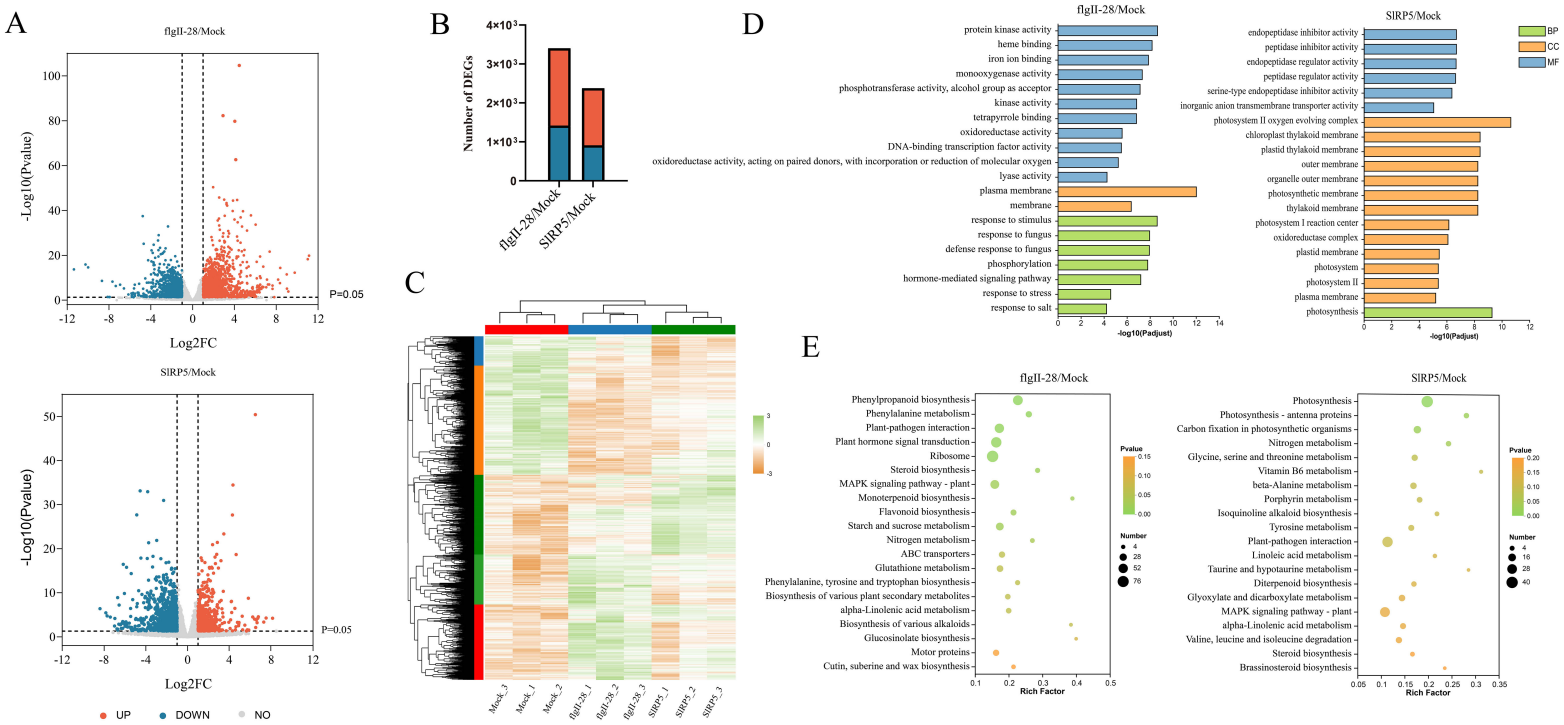
Transcriptomic analysis of tomato under SlRP5 and flgII-28 treatments. **(A)** Volcano plot depicting the DEGs under SlRP5 and flgII-28 treatments. Each dot represents a gene, with red dots indicating upregulated genes and blue dots indicating downregulated genes. **(B)** Number of DEGs identified in response to flgII-28 and SlRP5 treatments. **(C)** Hierarchical clustering of DEGs. The expression levels of each gene across different samples are displayed as Z-scores scaled from FPKM. **(D)** GO analysis of DEGs. **(E)** KEGG pathway analysis of DEGs.

To elucidate the downstream signaling pathways modulated by SlRP5, we performed a comprehensive analysis using transcriptomic sequencing (RNA-seq). Volcano plots revealed that transcriptional changes induced by flgII-28 were greater than those induced by SlRP5 (**Figure 4A**). Compared to the mock group, 3,413 genes exhibited significant differential expression following flgII-28 treatment (1,995 upregulated, 1,418 downregulated), whereas 2,386 genes were differentially expressed after SlRP5 treatment (1,475 upregulated, 911 downregulated) (**Figure 4B**). Heatmap analysis revealed a strong correlation among DEGs within each treatment group (**Figure 4C**). To further elucidate the functions of these DEGs, Gene Ontology (GO) annotation was performed, categorizing them into cellular components, biological processes, and molecular functions (**Supplementary Figure 2**). GO enrichment analysis revealed that DEGs from the flgII-28 treatment were primarily enriched in biological processes and molecular functions, whereas those from the SlRP5 treatment were mainly enriched in cellular components and molecular functions (**Figure 4D**; **Supplementary Table 3**). Additionally, KEGG analysis showed that DEGs from both treatments were commonly enriched in pathways such as plant–pathogen interaction, steroid biosynthesis, MAPK signaling pathway–plant, nitrogen metabolism, and alpha-linolenic acid metabolism (**Figure 4E**; **Supplementary Figure 3**; **Supplementary Table 4**).

**Figure 5:**
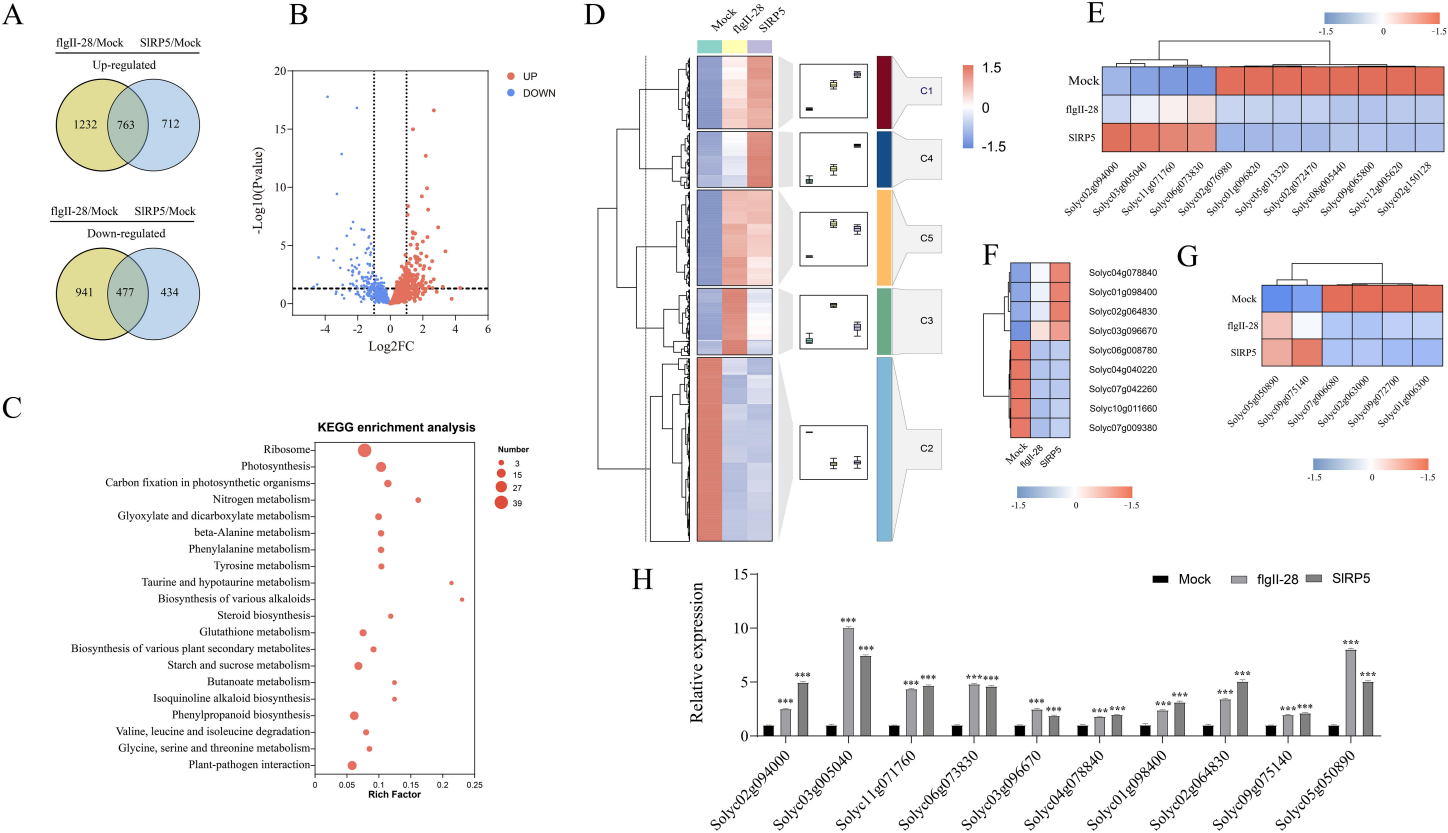
Comparative analysis of DEGs under SIRP5 and flgII-28 treatments. **(A)** Venn diagram showing DEGs in the comparison of flgII-28/mock and SIRP5/mock. **(B)** Volcano plot showing the relationship of common DEGS between SIRP5 and flgII-28 treat- ments. **(C)** KEGG analysis of common DEGs. **(D)** Clustering analysis of common DEGs using the Fuzzy C-Means algorithm (Mfuzz). **(E)** Relative expression levels of genes involved in the “plant-pathogen interaction” pathway. **(F)** Relative expression levels of genes involved in the “plant hormone signal transduction” pathway. **(G)** Relative expression levels of genes involved in the “phenylpropanoid biosynthesis” pathway. **(H)** RT-qPCR validation of the RNA-Seq transcriptomic data. Data are presented as means ± SEM (ANOVA, *n* = 4). Asterisks indicate a significant difference compared to the Mock : ***p < 0.001. The gradient color bar indicates gene expression values of normalized data on a scale from −1.5 to 1.5.

We analyzed the transcriptomic differences between SlRP5 and flgII-28 treatments. Compared to the mock group, SlRP5, and flgII-28 treatments shared 763 upregulated and 477 downregulated DEGs (**Figure 5A**). Further analysis showed that 668 genes were more highly expressed after SlRP5 treatment, while 570 genes were more highly expressed after flgII-28 treatment. The remaining genes exhibited similar expression levels under both treatments (**Figure 5B**). KEGG analysis revealed that these DEGs are enriched in several plant disease-related pathways, including “plant–pathogen interaction”, “plant hormone signal transduction”, and “phenylpropanoid biosynthesis” (**Figure 5C**; **Supplementary Table 5**). We performed clustering analysis on the expression patterns of these DEGs using the Fuzzy C-Means algorithm (Mfuzz) (**Figure 5D**). The analysis grouped the DEGs into five clusters. Genes in clusters 1 and 4 showed higher expression levels in the SlRP5 treatment compared to flgII-28, while genes in cluster 2 exhibited reduced expression in both treatments, with more pronounced suppression in the SlRP5 treatment. Specific gene analysis revealed that SlRP5 significantly upregulated four *CML* genes associated with plant–pathogen interaction (Solyc02g094000, Solyc03g005040, Solyc11g071760, and Solyc06g073830) (**Figure 5E**). Additionally, SlRP5 significantly upregulated nine genes related to plant hormone signal transduction, particularly Solyc03g096670, which encodes protein phosphatase 2C and was more prominently upregulated compared to flgII-28 (**Figure 5F**). In the “phenylpropanoid biosynthesis” pathway, six genes showed similar regulation, with Solyc09g075140 and Solyc05g050890 being more highly expressed after SlRP5 treatment than after flgII-28 (**Figure 5G**). These differential gene expressions likely reflect the distinct mechanisms by which SlRP5 and flgII-28 induce pathogen resistance. To validate the RNA-seq results, 10 genes from the plant–pathogen interaction, plant hormone signal transduction, and phenylpropanoid biosynthesis pathways were selected for qRT-PCR analysis. The expression patterns of these genes were generally consistent with the RNA-seq results (**Figure 5H**).

### 3.5 SlRP5 mitigates tomato damage from *B. cinerea* by enhancing antioxidant enzyme activity

**Figure 6:**
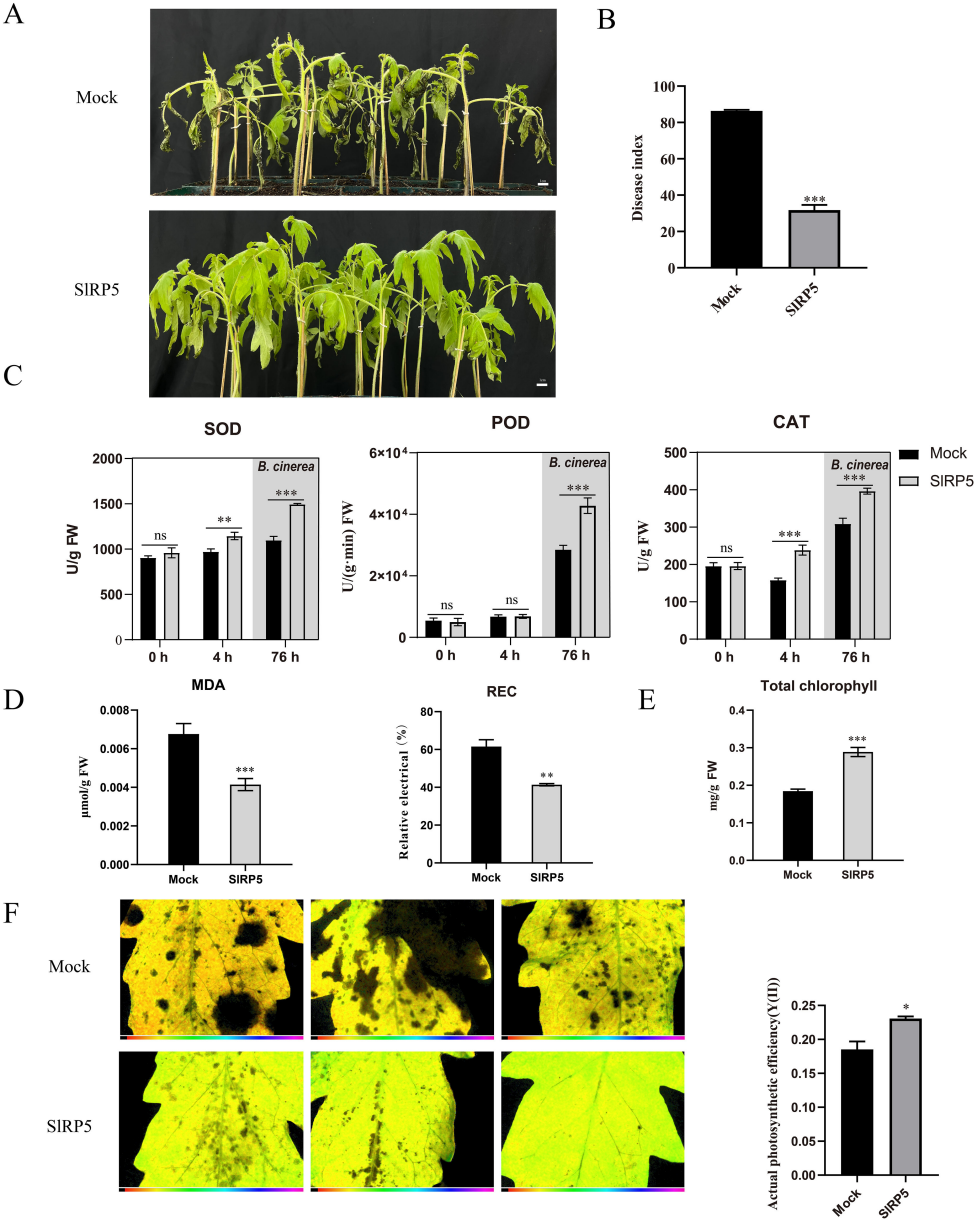
Protective effects of SIRP5 on tomato against *B. cinerea* infection. **(A)** Morphological changes in tomato leaves 72 hours after *B. cinerea* inoculation. **(B)** Quantification of disease index. Data are presented as means ± SEM (*t-test*, *n* = 3). **(C)** SOD, CAT, and POD activities in tomato leaves at different treatment time points. SOD, POD, and CAT activities were measured at and 4 hours after SIRP5 treatment. Subsequently, *B. cinerea* was introduced at 4 hours post-Sl- RP5 treatment, and the enzyme activities were measured again at 72 hours post-*B. cinerea* treatment. Data are presented as mean ± SEM (ANOVA, *n* = 12). **(D)** Analysis of MDA content and REC in tomato leaves at 72 hours post-*B. cinerea* treatment. Data are presented as means ± SEM (*t-test*, *n* = 3). **(E)** Total chlorophyll content in leaves 72 hours post-infection with *B. cinerea*. Data are presented as means ± SEM (*t-test*, *n* = 3). **(F)** Measurement of photosynthetic efficiency in leaves post-*B. cinerea* treatment using modulated imaging fluorometry (*t-test*, *n* = 3). Asterisks indicate significant differences compared to the Mock: *p < 0.05, **p < 0.01, ***p < 0.001. ns indicates no significant difference.

*B. cinerea* has a significant negative impact on tomato growth and development; however, SlRP5 pretreatment effectively mitigated this damage. As shown in **Figure 6A**, 72 h after *B. cinerea* inoculation, most leaves in the control group showed necrosis, whereas leaves treated with SlRP5 maintained a more intact morphology, significantly reducing the disease index (**Figure 6B**). To further investigate how SlRP5 enhances resistance to *B. cinerea* infection, we analyzed the antioxidant enzyme activity and stress-related physiological indices in tomato leaves. Results indicated that SlRP5 treatment significantly increased SOD and CAT activities after 4 h. Following *B. cinerea* inoculation and an additional 72-h infection period, we measured enzyme activity again, and the results showed that SlRP5 pretreatment significantly increased SOD, CAT, and POD levels, thereby enhancing plant protection (**Figure 6C**). Both biotic and abiotic stresses often lead to increased MDA and REC levels. We measured the changes in MDA content and REC 72 h after *B. cine*rea infection. As shown in **Figure 6D**, SlRP5 treatment significantly inhibited the rise in MDA and REC. *B. cinerea* infection can cause leaf tissue death and disrupt photosynthesis. Next, we measured chlorophyll content and photosynthetic efficiency postinfection. SlRP5 pretreatment partially preserved chlorophyll stability (**Figure 6E**). Using modulated imaging fluorometry, we found that Y(II) values were higher in SlRP5-treated leaves compared to the mock group, indicating that SlRP5 provided protection to photosynthetic function (**Figure 6F**). Collectively, these results demonstrate that SlRP5 can effectively protect tomatoes by mitigating the negative effects of *B. cinerea* on growth.

### 3.6 SlRP5 induces immune responses in other solanaceous crops

**Figure 7:**
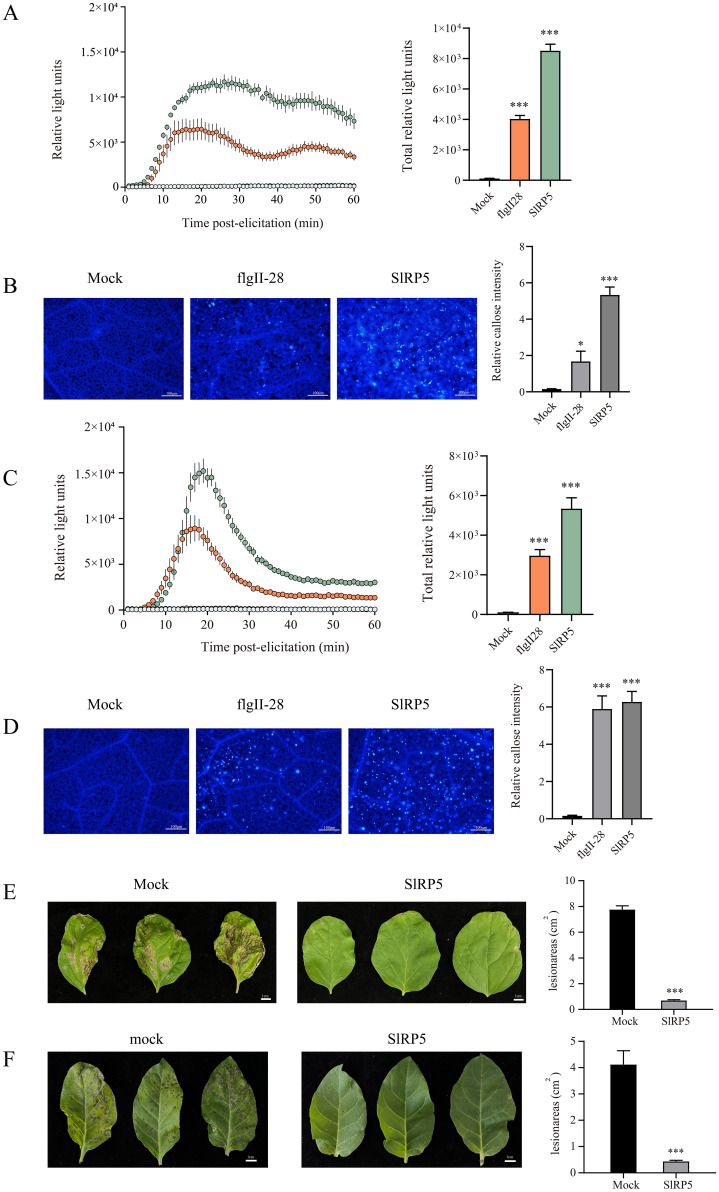
Effects of SIRP5 in inducing immune responses in Solanaceous crops. **(A)** ROS burst in eggplant leaves after treatment with SIRP5. The left panel shows the dynamics of ROS production over 60 minutes post-elicitation, while the right panel shows the total ROS production. Data are presented as means ± SEM (one-way ANOVA, *n* = 4). **(B)** Fluorescence microscopy images showing callose deposition in eggplant leaves treated with SIRP5. The right panel shows the relative intensity of callose deposition. Data are presented as means ± SEM (one-way ANOVA, *n* = 20). **(C)** ROS burst in tobacco leaves after treatment with SIRP5. The left panel shows the dynamics of ROS production over 60 minutes post-elicitation, while the right panel shows the total ROS production. Data are presented as means ± SEM (one-way ANOVA, *n* = 4). **(D)** Fluorescence microscopy images showing callose deposition in tobacco leaves treated with SIRP5. The right panel shows the relative intensity of callose deposition. Data are presented as means ± SEM (one-way ANOVA, *n* = 20). **(E)** Disease status of eggplant leaves inoculated with *B. cinerea* after treatment with SIRP5. The right panel quantifies the lesion areas. Data are presented as means ± SEM (*t-test*, *n* = 6). **(F)** Morphological changes in tobacco leaves infected with TMV after treatment with SIRP5. The right panel quantifies the lesion areas. Data are presented as means ± SEM (*t-test*, *n* = 6). Asterisks indicate significant differences from the Mock: *p < 0.05, ***p < 0.001.

SlRP5 demonstrated significant efficacy in inducing resistance against gray mold in tomatoes, prompting further investigation into its ability to trigger immune responses in other solanaceous crops. As shown in **Figures 7A–D**, SlRP5 induced ROS bursts and callose deposition in both eggplant and tobacco. Notably, the immune responses induced by SlRP5 were more robust than those triggered by the peptides alone. Following treatment with 3 μM SlRP5 for 24 h and subsequent inoculation with gray mold, eggplant showed significantly reduced pathogen damage (**Figure 7E**). In the tobacco experiment, we chose to study TMV instead of gray mold, as TMV is one of the most prevalent diseases in tobacco, causing significant losses in yield and quality, making it more representative and relevant. As shown in **Figure 7F**, tobacco leaves treated with SlRP5 exhibited significantly fewer symptoms compared to the control. Collectively, these results suggest that SlRP5 can bolster disease resistance in additional members of the Solanaceae family, including eggplant and tobacco, highlighting its potential for widespread application across Solanaceous crops.

## 4 Discussion

### 4.1 Comparison between SlRP5 and traditional peptide elicitors

In this study, we evaluated the role of 10 published peptides and SlPIP1 in tomato immune responses through ROS bursts. SlPIP1 is a biologically active peptide identified from the homologous identification of the PIP peptide family, which is known to elicit immune responses across various plant families, including Cruciferae, Solanaceae, Gramineae, Cannabaceae, and Leguminosae (Mersmann et al., 2010; Smith et al., 2014; Wei et al., 2020; Sands et al., 2022). Research on flgII-28, systemin, and csp22 has primarily focused on Solanaceae (Ryan and Pearce, 2003; Rosli et al., 2013; Ciarroni et al., 2018; Moroz and Tanaka, 2020). FlgII-28 demonstrates a potent capacity to induce immune responses in tomatoes, peppers, and potatoes (Clarke et al., 2013; Zeiss et al., 2021). This aligns with our findings that flg22, flgII-28, csp22, systemin, and SlPIP1 effectively trigger ROS bursts (**Figure 1A**).

Based on the established role of traditional peptide elicitors in plant immune responses, we developed a recombinant protein, SlRP5, which incorporates the active epitopes of flg22, csp22, flgII-28, SlPIP1 and systemin to enhance tomato immunity. Despite harboring multiple active epitopes, SlRP5 demonstrated relatively weak performance in triggering ROS bursts and activating MAPK phosphorylation, possibly due to its complex structural configuration. However, SlRP5 showed distinct advantages in inducing callose deposition and enhancing tomato resistance to *B. cinerea*, leading to a more pronounced effect on overall disease resistance (**Figure 3**). Notably, structural differences among single peptide elicitors can lead to significant variations in their ability to activate MAPK phosphorylation (Hind et al., 2016). For instance, flg22 can rapidly activate MAPK phosphorylation in *Arabidopsis* within 5 min, although this effect wanes after 30 min, while nlp20 induces a more sustained response. This disparity further underscores the unique role of SlRP5 in eliciting specific immune responses.

Previous studies have demonstrated that combining peptides, rather than using single peptides, can trigger more complex plant immune responses via partially overlapping yet independent signaling pathways (Zipfel et al., 2006). Treatment with a single peptide can elicit specific biological responses, such as ROS production, extracellular pH changes, and phosphorylation of certain proteins, up to a certain threshold. However, the addition of other peptides may initiate additional immune responses via independent pathways. Although SlRP5 did not elicit a more substantial ROS burst than individual peptides within the same timeframe, its integration of active epitopes from five different peptides likely facilitated cross-talk and integration of multiple immune pathways, thereby enhancing plant disease resistance. This mechanism of signal integration may explain why SlRP5 exhibits a more pronounced effect in resisting *B. cinerea* infection compared to single peptides.

Through detailed transcriptome analysis, we observed that both SlRP5 and flgII-28 activated similar KEGG enrichment pathways, such as plant–pathogen interaction and phenylpropanoid biosynthesis. However, SlRP5 induced a more significant expression of certain key genes. Specifically, SlRP5 treatment led to a significant increase in the expression of genes involved in calcium-binding proteins and the phenylpropanoid biosynthesis pathway, including Solyc02g094000, Solyc03g005040, Solyc11g071760, Solyc06g073830, Solyc09g075140, and Solyc05g050890. The upregulation of these genes may contribute to the enhanced defensive capabilities of SlRP5. Overall, SlRP5 is a recombinant protein that combines multiple active epitopes from plant peptides and is capable of activating plant immune responses.

### 4.2 The impact of SlRP5 on tomato resistance to *B. cinerea*

The jasmonic acid (JA) pathway plays a critical role in plant responses to pathogens, particularly in regulating defense mechanisms against various pathogens. Studies have shown that the accumulation of JA directly enhances plant resistance to pathogens (Coppola et al., 2015; Li et al., 2024; Liu et al., 2021). Additionally, certain microbial metabolites, such as oxalic acid secreted by *Bacillus* spp., have been demonstrated to significantly boost plant defense capabilities by activating the JA pathway (Yu et al., 2022). This finding further supports the strategy of using exogenous methods to activate the JA pathway as a means to enhance plant disease resistance. In this study, the expression of genes such as LoxD, MYC2, PI-I, and PI-II induced by SlRP5 treatment indicates that SlRP5 regulates tomato resistance to pathogens through the JA pathway.

Under normal growth conditions, the production and scavenging of ROS within plant cells maintain a dynamic balance (Wu et al., 2023). However, this balance is disrupted upon pathogen infection, leading to the excessive accumulation of ROS, which damages the integrity of plant cell membranes and causes oxidative damage. The antioxidant enzyme system plays a crucial role in combating biotic stress. Studies have shown that increasing the activity of antioxidant enzymes, such as CAT and POD, can effectively reduce ROS levels and enhance plant resistance to pathogens. In our study, SlRP5 pretreatment not only moderately increased the activities of SOD and CAT under nonpathogenic conditions but also further enhanced the activities of SOD, CAT, and POD after infection with *B. cinerea*, thereby strengthening the plant’s defense mechanisms (**Figure 6C**). Previous studies have shown that cell membrane damage is typically associated with lipid peroxidation, which can be effectively reflected by measurements of MDA and REC. In this study, we found that plants treated with SlRP5 exhibited significantly lower levels of MDA and REC after infection with *B. cinerea* compared to the untreated control group, indicating that SlRP5 effectively mitigates cell membrane damage caused by the pathogen (**Figure 6D**). Additionally, plants treated with SlRP5 demonstrated more effective retention of chlorophyll content and better maintenance of photosynthetic efficiency after infection with *B. cinerea* (**Figures 6E, F**).

### 4.3 The potential of SlRP5 as a broad-spectrum inducer of resistance in solanaceous plants

In modern plant disease management, microbe-associated molecular patterns (MAMPs) have garnered significant attention due to their ability to trigger broad-spectrum defense responses in plants. This function has been demonstrated in various species, including *Arabidopsis*, grape, tomato, and rice. These studies indicate that MAMPs are ideal candidates for developing new biopesticides. However, most existing MAMPs are produced through artificial synthesis, which significantly increases production costs and limits their widespread agricultural application. To address this challenge, our research focused on developing a novel recombinant protein, SlRP5.

SlRP5 not only exhibits significant immune-stimulating effects in tomatoes but also induces similar defense responses in other Solanaceae plants, such as eggplant and tobacco. Although capsicum was not included in the present study, the conserved nature of immune pathways across Solanaceae species suggests that SlRP5 may also trigger immune responses in capsicum. This hypothesis requires further validation to confirm SlRP5’s applicability to capsicum and other economically important crops. Future research should address this gap by evaluating SlRP5’s efficacy across a wider range of Solanaceae crops, including capsicum, under both experimental and field conditions. Additionally, future research should investigate the molecular mechanisms underlying SlRP5-induced immune responses in a broader range of Solanaceae crops, including capsicum, to confirm its applicability across this plant family. This broad-spectrum effect suggests that SlRP5 can activate widely conserved disease resistance mechanisms within plants, which is crucial for developing cross-species protection strategies. Moreover, this mechanism leverages the plant’s inherent defense potential rather than relying on external chemical substances, contributing to long-term plant health and sustainable agricultural production. Compared to traditionally synthesized peptides, producing SlRP5 using a prokaryotic expression system not only reduces production costs but also minimizes environmental impact. These findings highlight SlRP5’s potential as a cost-effective and environmentally friendly biopesticide for sustainable agricultural production. Although current research demonstrates that SlRP5 confers resistance to various Solanaceae plant diseases under experimental conditions, future studies should further explore its effectiveness under field conditions and investigate how to integrate this novel biopesticide most effectively into existing agricultural management systems.

## 5 Conclusion

This study successfully developed a recombinant protein, SlRP5, which integrates five active epitopes and demonstrated its efficacy in enhancing disease resistance in tomatoes and other crops. SlRP5 effectively triggered immune responses by inducing ROS bursts, MAPK phosphorylation, and callose deposition, thereby mitigating membrane damage caused by *Botrytis cinerea*. Additionally, SlRP5 exhibited similar immune-stimulating effects in tobacco and eggplant, enhancing resistance to *B. cinerea* in eggplant and TMV in tobacco. These findings establish SlRP5 as a promising candidate for the development of novel biopesticides, offering new strategies for integrated crop disease management.

## Data Availability

The datasets presented in this study are deposited in online repositories. The names of the repository and the accession number are as follows: https://www.ncbi.nlm.nih.gov/, PRJNA1219129.

## Supplementary material

Supplementary Table 1 Peptide information (xlsx).

Supplementary Table 2 Primer information (xlsx).

Supplementary Table 3 GO analysis of the DEGs in flgII-28 vs Mock and SlRP5 vs Mock (xlsx).

Supplementary Table 4 KEGG analysis of the DEGs in flgII-28 vs Mock and SlRP5 vs Mock (xlsx).

Supplementary Table 5 KEGG analysis of the DEGs in flgII-28 vs SlRP5 (xlsx).
