# Supplementary figures and images for: Engineering and application of multiepitope recombinant proteins to enhance resistance to Botrytis cinerea in tomatoes: a new paradigm for creating plant immune activators
Source: Front Plant Sci. 2025 Feb 17;16:1499777. doi: 10.3389/fpls.2025.1499777 (PMC11873852; doi:10.3389/fpls.2025.1499777)

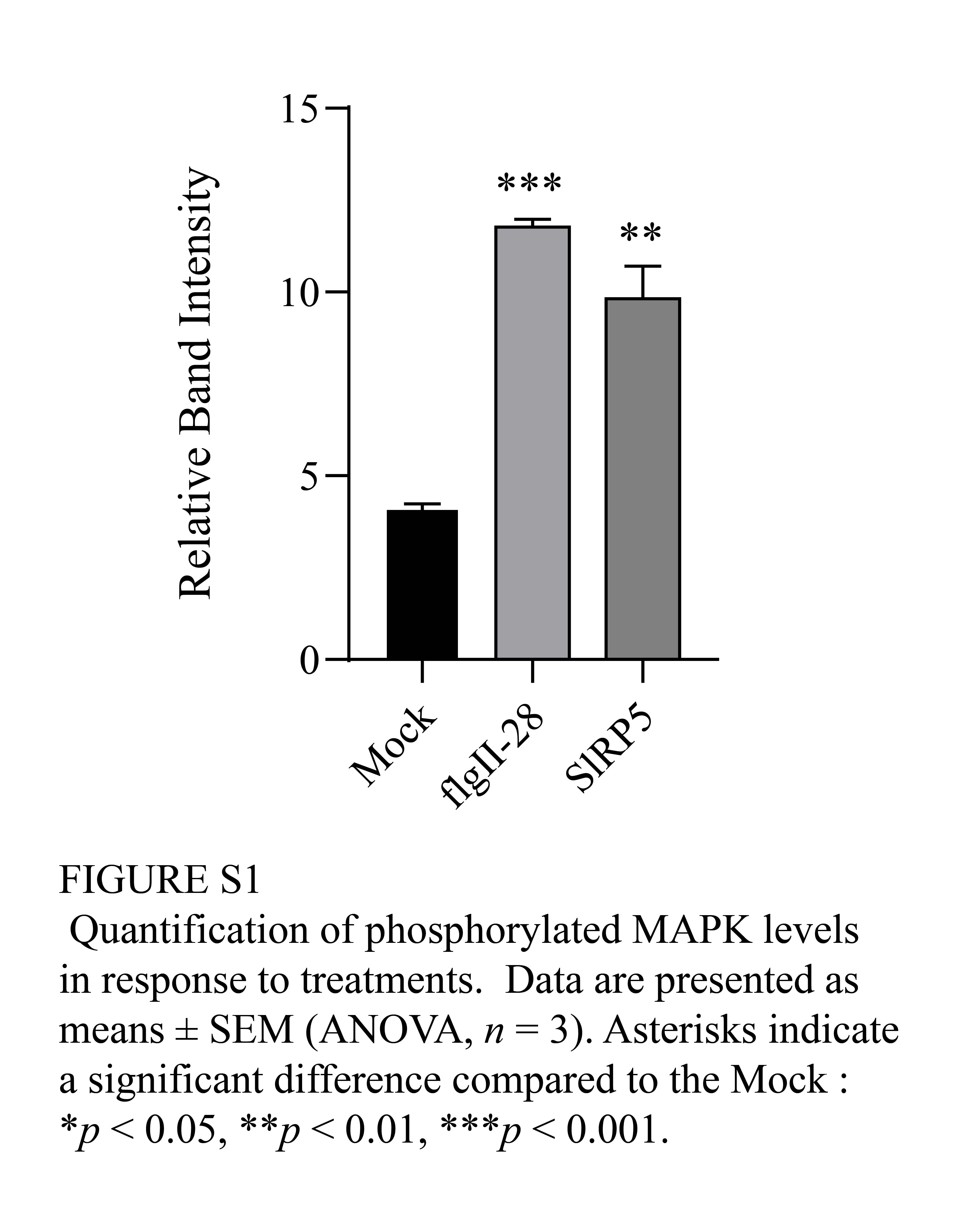

Supplement: Supplementary file 6 [file Image1.jpeg]

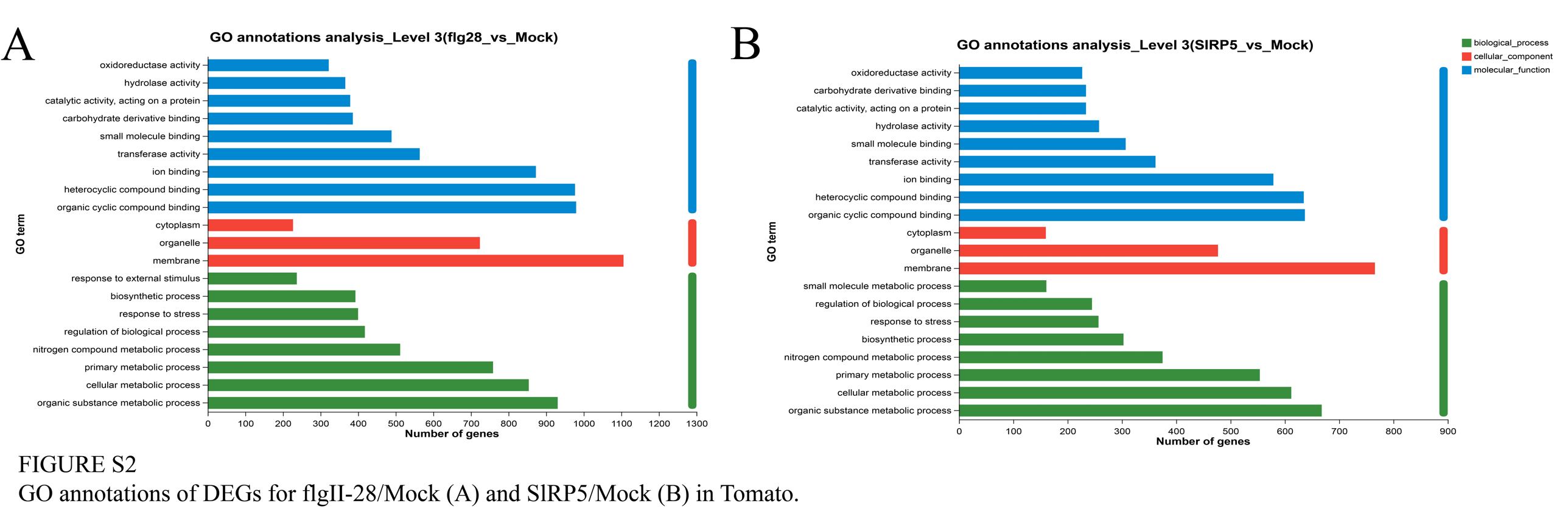

Supplement: Supplementary file 7 [file Image2.jpeg]

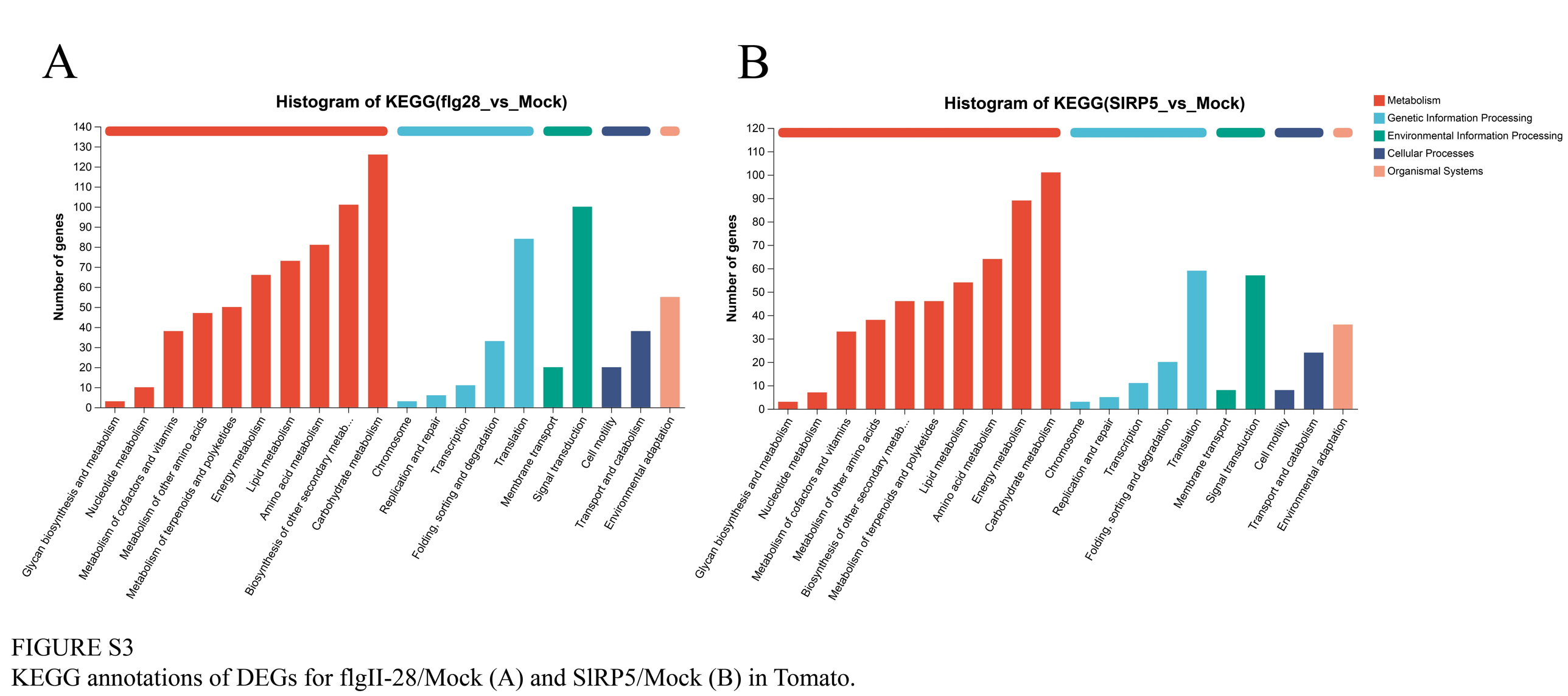

Supplement: Supplementary file 8 [file Image3.jpeg]
